# Philadelphia County Medical Society

**Published:** 1879-01

**Authors:** 


### PHILADELPHIA COUNTY MEDICAL SOCIETY.

A conversational meeting was held at the hall of the College of Physicians, Philadelphia, November 27, 1878, Dr. J. Solis Cohen, Vice-President of the Society, in the chair.

*The Question of Tracheotomy for Laryngeal Paralysis.—Relief of Asphyxia by Ice.*—Dr. M. O'Hara made some remarks upon a case in which the necessity for tracheotomy was averted by the systematic application of intense cold, in a child suffering with acute larygitis.

Dr. R. A. Cleeman referred to a case of capillary bronchitis in which anæsthesia of the nerve-centres was set up by the retention of carbonic acid in the blood, on account of the difficulty in getting air into the lung. The case was given up by the physician in charge, but the application of ice to the skin stimulated respiration, produced better aeration of the blood, and the child recovered.

Dr. O'Hara said that the urgency of the symptoms must be taken into consideration as one of the indications for tracheotomy. Although in the case reported the suprasternal and intrathoracic soft tissues sank in with each effort at inspiration, showing impediment, there was no obstruction to expiration, the blood received a fair quantity of oxygen, there were none of the signs of suffocation such as we see in capillary bronchitis, and there was no time when the blood was so deficient in oxygen as to require an operation. One of the great difficulties in regard to tracheotomy is the need of exact indications for the time of its performance; and the case was reported as a contribution to this subject.

Dr. Nancrede agreed with the lecturer, and stated that where the difficulty in breathing only comes on at intervals and in paroxysms, tracheotomy is not indicated; but where there is manifestly less air rushing into the chest, and the obstacle to its ingress becomes permanent, if the state of the child otherwise warranted the operation, he thought that such cases are just the ones for tracheotomy, which should not be eferred until the child is exhausted.

Dr. W. R. D. Blackwood said that the subject of tracheotomy is always one of great interest, but probably there is no other operation in surgery about which more diverse views of its utility have been expressed. One explanation of such a doubt may be found in the fact, as suggested by Dr. Nancrede, that it is generally performed too late. He had had two cases of tracheotomy in this city, and had assisted in several others. The importance of early operation is not sufficiently dwelt upon, and it should be brought more prominently before the profession. Physicians are inclined to wait until all other resources have been tried and failed. In his first case, one of membranous croup, he had fully determined to operate as soon as symptoms of suffocation came on, but the family then objected, because the child looked so well: when he got manifestly worse they consented, but it was too late. He was satisfied that if performed earlier it would have been successful; the child lived only a few minutes after the operation. The second case recovered after tracheotomy.

The application of cold in this connection is new, but the fact of its stimulating power in other cases is well known. In chloroform-narcosis, a piece of ice in the rectum will produce inspiration after breathing has stopped, and ice in the vagina will produce reflex contractions of the uterus.

Probably the case reported would have recovered under other treatment as well. There could not have been a closure of the glottis; for in that event, if there was no room for it, no amount of stimulation would have allowed the air to get past. If the trachea were closed, the application of the cold would have done but little good.

Dr. O'Hara believed the trouble to be due to paralysis of the muscles that dilate the glottis. The application of cold overcame this, just as water thrown on the face of a new-born child causes deep inspiration.

Dr. Nancrede said that if there were actual paralysis of the glottis, strong inspirations would increase the dyspnœa, by sucking in the valve-like cords, while gentle breathing would not disturb them. On the other hand, complete paralysis would not be relieved by the application of cold.

Dr. O'Hara said that Dr. Chapman, in his writings on the effects of the application of ice to the spine, which he had seen since writing his paper, also refers to the use of ice in croup and other diseases. The result in his case must have been due to the reflex action of the cold upon the brain. There is no doubt that this case recovered without a severe operation, and that an operation might have been the cause of his death. More precise rules for tracheotomy should be formulated from the teachings of experience.

Dr. Nancrede, having operated upon a number of cases, spoke from experience, in saying that paroxysmal dyspnœa was not an indication for operation so much as permanent laryngeal obstruction.

The Chairman had seen this case, which was a very interesting one, and believed it to be just the kind that had been referred to by Dr. Nancrede. There was no obstruction to expiration, but the stridor kept getting worse and worse. He had seen the same in adults, and Niemeyer speaks of seeing it in children, and considers it part of the history of croup. In adults there is nothing to be done but tracheotomy, which must be performed sooner or later, or the patient will perish.

The child he had been called in to see in consultation by Dr. O'Hara was suffering from the difficulty described, but as it was in apparent good condition, although just recovering from scarlet fever, he hesitated to recommend tracheotomy. Immediately upon the application of the ice to the neck, he took a deep inspiration. This artificial respiration was kept up for more than thirty hours, as mentioned, there being no deposit in the larynx and no obstruction to expiration.

He had another case in an adult recently in the same condition, but ice had no effect. Many of those cases where death from croup has occurred without deposit in the larynx, are precisely like the present, and due to paralysis of the dilator muscles of the glottis. The device of putting the cloths between two blocks of ice is a good one, as it keeps them cold all the time.

In reply to a question from Dr. O'Hara, he stated that he never had had the courage to use electricity in this form of paralysis, for fear of exciting spasm of the glottis and killing the patient.

*Compound fracture of anatomical neck of humerus.*—Presented by Dr. C. B. Nancrede.

This specimen, and the case from which it was removed, have seemed of such unusual interest as to induce me to offer them for your consideration this evening. Similar injurles, or rather injuries requiring similar treatment, are not unusual in military practice, but are very rarely met with elsewhere. I will first show the specimen, next describe the injury, and then exhibit the results, after which I will explain my reasons for the course pursued. The portion which I hold in my hand is, as you see, the upper four and a quarter inches of the diaphysis of the left humerus, completely stripped of all muscular attachments and periosteum, except at its posterior aspect, where a triangular slip remains, a portion of it being very thin, evidently consisting of only a few of the deeper layers. This portion is the upper epiphysis, the line of fracture passing through the epiphyseal line except at one or two points. The shaft is slightly split, while the shell of bone connected with the head is fissured at various portions of its circumference, as if the shaft had been impacted, thus wedging it apart. Further description of the specimen is involved in the desciption of the case, which I will now proceed to give.

The patient, J. McC., a boy aged 14 years, fell from a tree, some twenty-five feet, upon his elbow, on the afternoon of Sunday, September 23, 1878, landing on the ground, striking in his fall against the limbs of the tree, and sustained the following injuries in addition to those now detailed. The broken shaft was driven through the skin covering the lower portion of the deltoid muscle on its anterior aspect, tearing in its course the following parts. The insertion of the deltoid was completely stripped off with the subjacent periosteum; the coraco-brachial, teres major, and latissimus dorsi were in like manner torn off, the latter carrying with them the posterior lip of the bicipital groove. The tendon of the pectoralis major was torn off about half an inch from its insertion, and one if not both heads of the biceps were ruptured. In consequence the head and neck of the bone, deprived of periosteum, merely hung suspended from the glenoid cavity by its capsular ligament and the rotator muscles. The upper end of the lower fragment must have almost grazed the brachial artery, its point of emergence through the skin being not more than about three-fourths of an inch from its course. As you see, the patient now, about ten weeks since his injury, has a surprising amount of movement of his new joint. Seven weeks after the operation he removed his coat, vest and shirt without assistance. He can readily put his hand to his mouth, behind his back, and on his ear. He enjoys perfect use of his forearm, but of course has lost nearly all power of lifting the arm from the side. All of these motions are unattended with pain. Although four and a half inches by actual measurement of the bone have been removed, the actual shortening only amounts to a scant inch and a half, the new joint having formed apparently on the third rib.

The course of treatment pursued, and my reasons for deciding upon it, seem worthy of detail, since such injuries are but seldom seen, and, as far as I can discover, no clear rules have been laid down for their treatment. To the members of this Society who devote themselves especially to surgery I need hardly say that no question of amputation arose in my mind; but to those in pure medical practice I would say that when the main vessels and nerves of a limb remain intact, the injury to the soft parts having been produced by the bone itself, not the fracturing force, almost any degree of shattering of the bones may be recovered from in the young without operation. Two lines of treatment then offered for consideration, viz.: the return of the bone, closure of the skin-wound, drainage, and trusting the case to nature, or the resection of the injured bone. Theoretically the first would have seemed the better course, promising no shortening of the limb, and the retention, in a measure, of the power of the deltoid. In reality, however, the chances of union were not one in a thousand, and if not union then necrosis with its consequent shortening; necrosis, too, meaning months or years of inflammation and suppuration, matting the muscles together so that when recovery occurred—almost necessarily by an operation—the usefulness of the limb would be but slight. Resection, on the other hand, offered the complete removal of all injured portions of bone, and with them the most important factors of trouble after such an injury, thus permitting rapid healing, and the least possible inflammatory adhesions of the muscles, tendons, etc. If the bone had been simply returned, the risk to life would have been greater, owing to the prolonged suppuration incident upon the separation of the necrosed bone and deep-seated abscesses so common after compound fractures. Against it was the absolute shortening of the arm, with the prospective cessation of growth due to the removal of one of the humeral epiphyses.

The actual result, I think, bears me out in the course of treatment pursued, for I hardly think that he would be here in as good condition, with the wound soundly healed, if I had followed what is often, but falsely, called the "conservative plan" of treatment. I believed that true conservatism indicated exactly what I did. The amount of shortening would not have been much less had tne case been left to nature and necrosis. Had this occurred, union of the severed head could hardly have taken place; and then the same shortening would have obtained as surely as if the epiphysis had been removed. Army experience has shown that when a portion of the upper end of the humerus is removed for injury, nothing is gained by leaving the uninjured head, since it necroses. Although not cognizant of this fact of experience at the time of operaton, anatomical knowledge, general surgical principles, and experience induced me to arrive at a conclusion by a priori reasoning which I have since found that extended experience had already proved. I believe, therefore, that theoretically and from experience, resection ought to be performed for such injuries. It is hardly necessay to say anything about the operation itself, since each case must be a rule for itself, the only point being to remove the bones with as little additional damage to the soft parts as practicable. The wound was dressed antiseptically, and when I transferred the wards to my colleague, Dr. Packard, no suppuration had occurred, and there was not the slightest inflammatory blush about the wound. He did uninterruptingly well, and the wound was soundly healed in less than seven weeks, the greater part at a much earlier date, however.

Dr. Charles T· Hunter believed that there had been positive reproduction of the bone. The shaft extends to lower margin of glenoid cavity, and he recognized the insertion of the pectoral and latissimus dorsi muscles. If the bone had been restored to its place, he believed that the periosteum would have regained its attachment; there is no doubt that the head ought to have been removed, as its nourishment by the anterior circumflex artery was cut off.

Dr. Nancrede said that the idea of saving the bone had occurred to him, but he had adopted what seemed at the time of the accident to offer the best results, in view of the possibility of necrosis.

*Treatment of fractures of Humerus.*—Dr. J. Levis said that the case had been well treated, and the result was excellent. He had for a long time considered the ordinary routine treatment of fractures of the humerus in the vicinity of the shoulder as very unsatisfactory. The displacement ordinarially is of the upper fragment outwards. Representing, by a diagram, a fracture in the upper third, we find that the arm is shortened by the overlapping of the fragments. This shortening is easily overcome by carrying the elbow out from the body and making extension,—that is, bringing the lower fragment in a line with the upper one, which is tilted outwards. Keeping the fragments in this relation suggests the principle of treatment. In a case of this kind, recently, the patient had been treated only by the postural method,—lying in bed with his arm at right angles to his body. Surgeons are too apt to cover up such fractures with a cap and splints.

In the case of a colored woman, he had obtained good apposition by placing the hand upon the opposite shoulder, and in another case the forearm had to be placed behind the back. He had, for a number of years, discarded splints in treating fractures of the upper extremity of the humerus.

In these cases the displacement is due to the direction of the fracture, but there is no uniformity in the line of fracture or displacement. Each case is a rule for itself in the indications for treatment. His observation had led him to believe that fractures of the upper extremity of the humerus should be treated without splints by the postural treatment, using adhesive plaster to keep the arm across the body in the most favorable position; and possibly a pad may be needed in the axilla. By this means he was confident that better results can be obtained than by the usual practice.—*Medical Times.*

